# Treatment with the vascular endothelial growth factor-A antibody, bevacizumab, has sex-specific effects in a rat model of mild traumatic brain injury

**DOI:** 10.1177/0271678X231212377

**Published:** 2023-11-07

**Authors:** Mujun Sun, Tamara L Baker, Campbell T Wilson, Rhys D Brady, Glenn R Yamakawa, David K Wright, Richelle Mychasiuk, Anh Vo, Trevor Wilson, Josh Allen, Stuart J McDonald, Sandy R Shultz

**Affiliations:** 1 Department of Neuroscience, 2541 Central Clinical School, Monash University, Melbourne, VIC, Australia; 2 Monash Health Translation Precinct, 2541 Monash University, Melbourne, VIC, Australia; 3 Health Sciences, 5691 Vancouver Island University, Nanaimo, BC, Canada

**Keywords:** Avastin, cognition, concussion, neuroinflammation, VEGF-A antibody

## Abstract

Mild traumatic brain injury (mTBI) involves damage to the cerebrovascular system. Vascular endothelial growth factor-A (VEGF-A) is an important modulator of vascular health and VEGF-A promotes the brain’s ability to recover after more severe forms of brain injury; however, the role of VEGF-A in mTBI remains poorly understood. Bevacizumab (BEV) is a monoclonal antibody that binds to VEGF-A and neutralises its actions. To better understand the role of VEGF-A in mTBI recovery, this study examined how BEV treatment affected outcomes in rats given a mTBI. Adult Sprague-Dawley rats were assigned to sham-injury + vehicle treatment (VEH), sham-injury + BEV treatment, mTBI + VEH treatment, mTBI + BEV treatment groups. Treatment was administered intracerebroventricularly via a cannula beginning at the time of injury and continuing until the end of the study. Rats underwent behavioral testing after injury and were euthanized on day 11. In both females and males, BEV had a negative impact on cognitive function. mTBI and BEV treatment increased the expression of inflammatory markers in females. In males, BEV treatment altered markers related to hypoxia and vascular health. These novel findings of sex-specific responses to BEV and mTBI provide important insights into the role of VEGF-A in mTBI.

## Introduction

Mild traumatic brain injury (mTBI) has an estimated annual incidence of 64–74 million worldwide.^1^ mTBI often results in debilitating short-term symptoms, and there is evidence that >50% of mTBI patients experience persisting symptoms for months to years after the injury.^2–4^ mTBI is also a risk factor for the later development of neurodegenerative conditions such as dementia,^5^ Parkinson’s disease,^6^ and chronic traumatic encephalopathy.^7^ Despite the high incidence of mTBI and risk of poor outcomes, there are limited intervention options that improve mTBI recovery.^8^ This is in part due to knowledge gaps pertaining to the underlying pathophysiology, and the influence of modifying factors such as biological sex.

The cerebrovascular system supplies oxygen and nutrients to the brain that are vital to its function. Damage to the cerebrovasculature due to the forces occurring at the time of mTBI, as well as secondary injury cascades that involve cerebral blood flow (CBF) changes, perivascular neuroinflammation, and blood brain barrier (BBB) impairments, may all contribute to the symptomatic aftermath of mTBI.^9–14^ For example, there is evidence from both preclinical and clinical studies indicating that a single mTBI can result in BBB damage, CBF abnormalities, and neuroinflammation.^10,15–20^ There is also some evidence that cerebrovascular impairments after mTBI differ between males and females.^21,22^

Vascular endothelial growth factor-A (VEGF-A) is a potent promoter of angiogenesis and an essential regulator of vascular health.^23,24^ Upregulation of VEGF-A is neuroprotective in animal studies of severe TBI, enhancing microvascular repair, neurogenesis, and functional recovery.^25,26^ Bevacizumab (BEV), a USA FDA approved cancer treatment, is an anti-VEGF-A monoclonal antibody that binds to VEGF-A and neutralizes its actions.^27^ A previous rat study found that treatment with BEV resulted in worse brain damage and neurological deficits after a severe TBI.^28^ With that said, the role of VEGF-A in mTBI is not well characterized, with conflicting results to date. For example, one of the few effective interventions for mTBI patients is exercise,^29^ which is known to to upregulate VEGF-A in the brain.^30,31^ However, a recent study from our laboratory found that immediate and continuous treatment with VEGF-A did not improve subacute recovery in either male or female rats given an mTBI, with some evidence that it was actually detrimental.^32^ Therefore, the aim of the present study was to further our understanding of the role of VEGF-A in mTBI by blocking its activity with BEV in female and male rats.

## Material and methods

### Animals

Sprague-Dawley rats (42 males and 42 females) were obtained from the Monash Animal Research Platform. Power calculations were performed using G*Power (v3.1) to determine the sample size required to achieve alpha = 0.05 and power = 0.8. Based on effect sizes (0.45-0.55) in our previous studies using the same outcomes, 41 rats/sex were required for behavior and 29 rats/sex were required for gene expression. Rats were housed under a 12:12 light/dark cycle, with access to food and water *ad libitum*. Rats were group housed until cannula implant, at which point they were single housed for the remainder of the study. The rats were 10 weeks old at the time of injury. All procedures were approved by the AMREP Animal Ethics Committee (E/2029/2020/M) and were conducted in fulfilment of the requirements of the Australian Code of Practice for the Care and Use of Animals for Scientific purposes. The study followed the ARRIVE guidelines for reporting animal experiments.

### Experimental groups and study design

Rats were randomly assigned to their experimental groups using an online random generator (random.org). Rats were implanted with a cannula in the lateral ventricle of the brain at nine weeks of age. One week after the cannula implantation, rats were randomly assigned to receive either a mTBI via the lateral impact (LI) model or a sham injury and either BEV (Avastin®, Roche) or vehicle (VEH) treatment (artificial cerebrospinal fluid, ACSF; In Vitro Technologies). As such, there were four experimental groups: Sham + VEH (n = 10/sex), Sham + BEV (n = 10/sex), mTBI+VEH (n = 11/sex), mTBI + BEV (n = 11/sex). Behavioral testing was conducted over the subsequent ten days, and rats were euthanized 11 days after the mTBI for tissue collection and post-mortem analysis. All experiments were performed by researchers blinded to the condition.

### Intracerebroventricular (ICV) cannulation and drug administration

Anesthesia was induced with 4% isoflurane and maintained at 2% isoflurane throughout the cannula implantation surgery. The cannula (Alzet®, brain kit 2) was implanted into the lateral ventricle at the following coordinates relative to the bregma: posterior 0.2 mm, lateral 1.6 mm and at 4.5 mm depth from skull surface.^25,32–34^ The cannula was stabilized to the skull using dental cement and two stainless-steel screws inserted into the skull. The cannula tubing was sealed, and the incision on the head was sutured. Analgesia (buprenorphine, 0.05 mg/kg) was delivered during surgery, and again at 12- and 24-hours after surgery. The rat was placed on a heating pad during the ICV cannulation surgery to maintain normothermia.

One week after ICV cannulation, a mini osmotic pump (Alzet®, model 2002) was implanted subcutaneously between the scapulae under anesthetic and connected to the cannula.^35^ The osmotic pump then delivered either the VEH (with bovine serum albumin, BSA) or BEV (10 mg/kg in VEH with BSA) at a rate of 0.5 µL per hour for the duration of the entire study. This dose was chosen based on previous rodent brain insult studies that involved BEV treatment.^28,36^ The BSA (Sigma A4919) was added at 1 mg/mL as carrier proteins.^25^ The rat was placed on a heating pad during the pump implantation surgery to maintain normothermia.

### The LI model of mTBI

Whilst still under anesthetic after the pump implantation, the rat underwent a LI-induced mTBI or a sham injury.^32,37,38^ Rats were placed sternally in the device on a Teflon® board, with the left temporal lobe area positioned against the impactor. The impactor propelled a 50 g weight using pneumatic pressure at 14 m/s (∼137 G) into a helmet-like metal plate (30 × 13 × 3mm^3^), resulting in the acceleration of the rat head and a 180° lateral rotation. Consistent with clinical mTBI, a single LI does not result in macroscopic structural brain damage assessed via conventional neuroimaging (e.g., structural MRI);^38^ however it does induce neuroinflammation, axonal injury, and behavioral deficits.^32,39,40^ The rat was then removed from the device, and the rat’s self-righting reflex time and body weight was recorded (Table 1). Sham-injured rats underwent the identical procedures (i.e., ICV cannulation, pump implantation, anesthesia, placed in LI chamber), except that the LI was not induced. After the injury, the rat was again placed on a heating pad during the acute recovery to maintain normothermia.

**Table 1.** Self-righting times and body weight at the time of mTBI.

	Sham + VEH	Sham + BEV	mTBI+VEH	mTBI + BEV
Male				
Self-righting (sec)	155.0 [121.3, 224.8]	205.5 [153.0, 246.3]	293.0 [230.0, 381.0]*	291.0 [166.0, 313.0]*
Body weight (g)	319.9 ± 33.5	318.4 ± 27.5	318.8 ± 30.2	324.2 ± 22.6
Female				
Self-righting (sec)	152.5 [105.5, 195.0]	167.5 [110.5, 220.5]	265.0 [220.0, 380.0]*	240.0 [194.0, 491.0]*
Body weight (g)	208.5 ± 17.1	212.1 ± 13.1	209.3 ± 13.7	211.5 ± 15.0

Within each sex, the mTBI groups had significantly longer self-righting reflex times (median [the 25th percentile, the 75th percentile]) than the Sham groups. Within each sex, there were no statistically significant differences between the VEH and BEV groups for self-righting times or between any of the groups for body weight (mean ± SD; *p* > 0.05). * = mTBI greater than SHAM, *p* < 0.05.

## Behavioral testing

### Elevated plus maze (EPM)

One day after mTBI, rats underwent EPM testing to examine anxiety-like behavior.^41,42^ The apparatus consisted of 4 cross shaped arms (two opened arms and two closed arms, each 50 × 10 cm). The closed arms were shielded by walls 30 cm high. The rat was placed in the center of the maze facing an open arm and allowed to explore the maze freely for 5 minutes. The rat’s movement was tracked by TopScan^TM^ 3.0 (Clever Sys., Inc., USA). A percentage score was calculated as time in the open arms/(time in the open arms + time in the closed arms) as a measure of anxiety-like behavior.^41,43,44^

### Open field

Two days after mTBI, the rats underwent open field testing.^42,45^ The apparatus consisted of a circular open arena (100 cm diameter) surrounded by 30 cm high walls. An inner area (66 cm diameter) was defined as the middle field. The rat was placed into the center of the arena and allowed to explore for 5 minutes. The rats were tracked using TopScan^TM^ 3.0 software which measured time spent in the middle field (levels of anxiety) and total distance travelled (general locomotor activity) in the arena.

### Beam

One week after mTBI, rats were tested on the beam task to assess sensorimotor function.^42,46^ Rats were first habituated to the beam (100 cm long) the day prior to testing. This required the rat to traverse a 4 cm wide beam five times, which was followed by traversing a 2 cm wide beam five times. Beam testing involved 10 trials on the 2 cm wide beam. A maximum time of 60 seconds was allowed for the rat to cross the beam. The time taken for each rat to traverse the beam, as well as the number of slips and falls were recorded by an observer blinded to the condition.^46^ Slips were defined as any limb that slipped from the beam.

### Water maze

One week after mTBI, rats underwent water maze testing.^41,42,46,47^ The water maze apparatus is a circular pool (174 cm diameter) filled with water. The test was performed under dim light. A hidden platform (10x10cm^2^) was submerged 3.5 cm below the surface of the water to provide the rats a means to escape from the water. Four visible cues were fixed around the pool perimeter for spatial orientation. Rats completed 10 trials each day, over two days. On the first day (i.e., the acquisition session), the platform was placed in the south-east quadrant. On day two (i.e., the reversal session) the platform was moved to the diagonally opposite quadrant. Each trial began at one of four randomized start locations around the perimeter of the pool (i.e., north, south, east, and west), where the rat was released facing the wall. All rats were given a maximum of 60 seconds to explore the arena and locate the platform. If the rat did not locate the platform, they were led to the platform by the researcher. After 15 s on the platform, the rat was removed from the pool, towel dried, and placed under a heating lamp. The rat’s movement was tracked with TopScan^TM^ 3.0 which calculated search time to reach the platform and swim speed. Direct and circle swims, defined as a swim path to the platform from the start point without any crossing swims or with an arc not exceeding 360°, was also calculated.

## Gene expression analysis

Rats were euthanized on day 11 post-injury. The ipsilateral hippocampus and ipsilateral temporal cortex were dissected, rapidly frozen, and stored at –80 °C until analysis. These regions are known to be affected by the LI model and/or are relevant to the behavioral outcomes examined.^48^ Total RNA (n = 7–9/sex/group) was isolated from 20 mg tissue by a RNeasy® Mini Kit (Qiagen) running by QIAcube (Qiagen). 200 ng yielded RNA proceeded to cDNA synthesis using Quantabio qScript XLT cDNA SuperMix following instructions from the manufacturer (Quantabio). Multiplex qPCR was performed with Fluidigm BioMark HD™. For each sample, 1.25 μL of the resulting cDNA were combined with 3.75 μL of Sample Pre-Mix (Life Technologies TaqMan® PreAmp Master Mix and Pooled Taqman assays) and preamplified for 14 cycles. The reaction products were diluted 1:5 and loaded onto the Gene expression IFC according to Fluidigm® IFC Standard Taqman Gene expression workflow. 42 TaqMan® gene expression assays related to neuroinflammation, hypoxia injury and vascular health, and 4 housekeeping gene assays were used and detailed in Supplementary Table 1. All hippocampal samples were analyzed simultaneously on a single Multiplex qPCR plate, and all temporal cortex samples were analyzed simultaneously on a single Multiplex qPCR plate. Cycle threshold (Ct) values were collected for analysis, using the 2^−ΔΔCt^ method.^49^

## Statistical analysis

Data were analyzed with SPSS 29.0 software (IBM Corp, Armonk, USA), and statistical significance was set at *p* < 0.05. The primary objective of the study was to investigate the impact of BEV treatment within each sex (i.e., it was not designed or statistically powered to directly examine between sex differences); therefore, female and male data were analyzed separately. The Shapiro–Wilk test normality test was performed to assess the data distribution, and data were assessed by 2-way ANOVA (treatment and injury as between-subject factors, followed by Bonferroni post-hoc comparisons when appropriate) or Kruskal-Wallis test accordingly. The gene expression data were transformed by log_2_ and analyzed by 2-way ANOVA. Water maze search time was analyzed by mixed-design analysis of variance (ANOVA), with treatment (BEV, VEH) and injury (sham, mTBI) as between-subjects variables and day as the within-subjects variable.

## Results

### Behavior findings in female rats

**Figure 1.**
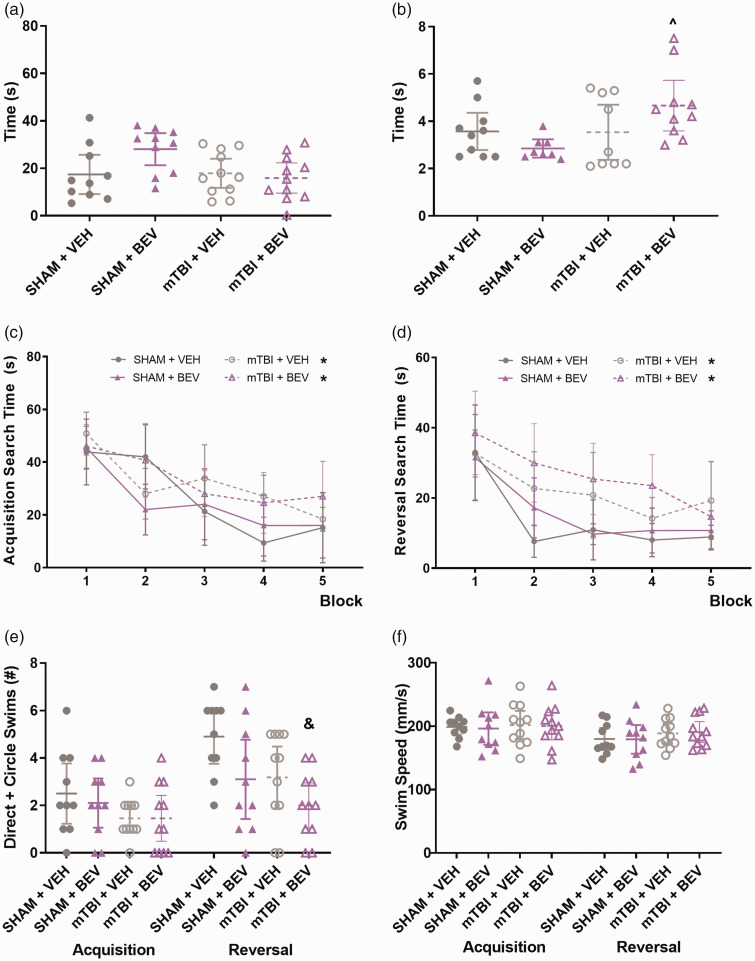
Behavior findings in female rats. No statistically significant findings were found on the measure of time spent in the middle of the open field (a). mTBI + BEV rats took longer to traverse the beam than the Sham + BEV rats (b). mTBI rats spent significantly longer time than sham rats to find the platform during both acquisition and reversal sessions (c&d). mTBI + BEV rats had fewer direct and circle swims than the Sham + VEH rats during reversal (e). No significant differences were found on swim speed (f). ^mTBI + BEV significantly differed from Sham + BEV, *mTBI significantly differed from sham, ^&^mTBI + BEV significantly differed from Sham + VEH; data are presented as mean with 95% CI; block = mean of every 2 trials.

On the measure of time spent in the middle of the open field, there was a statistically significant finding (H (3) = 8.926, *p* < 0.05; Figure 1(a)), however no significant differences were found between the groups with post-hoc comparisons (*p* > 0.05).

On the measure of traverse time on the beam test, there was a significant difference between the groups (H (3) = 7.821, *p* < 0.05; Figure 1(b)) and post-hoc analysis found that mTBI + BEV rats took longer to traverse the beam than the Sham + BEV rats (*p* < 0.05).

During water maze acquisition, there was a significant main effect for injury (F_1,38_ = 5.212, *p* < 0.05; Figure 1(c)) on the measure of search time, with mTBI rats taking significantly longer than sham rats to find the platform. There was also a significant main effect for trial (F_9,342_ = 16.853, *p* < 0.0001; Figure 1(c)) on the measure of search time, with rats spending less time to find the platform as testing progressed. No significant findings were found on the measures of direct and circle swim or swim speed (*p* > 0.05; Figure 1(e) and (f)).

During water maze reversal, there was a significant main effect for injury (F_1,38_ = 14.922, *p* < 0.0001; Figure 1(d)) on the measure of search time, with mTBI rats spending significantly longer time than sham rats to find the platform. There was also a significant main effect for trial (F_9,342_ = 15.907, *p* < 0.001; Figure 1(d)) with rats improving as testing progressed. For the measure of direct and circle swims, there was a statistically significant difference between the different groups (H (3) = 10.658, *p* < 0.05; Figure 1(e)) and post-hoc comparisons found that mTBI + BEV rats had fewer direct and circle swims than the Sham + VEH rats (*p* < 0.05). No significant findings were found on the measure of swim speed (*p* > 0.05; Figure 1(f)).

### Behavior findings in male rats

**Figure 2.**
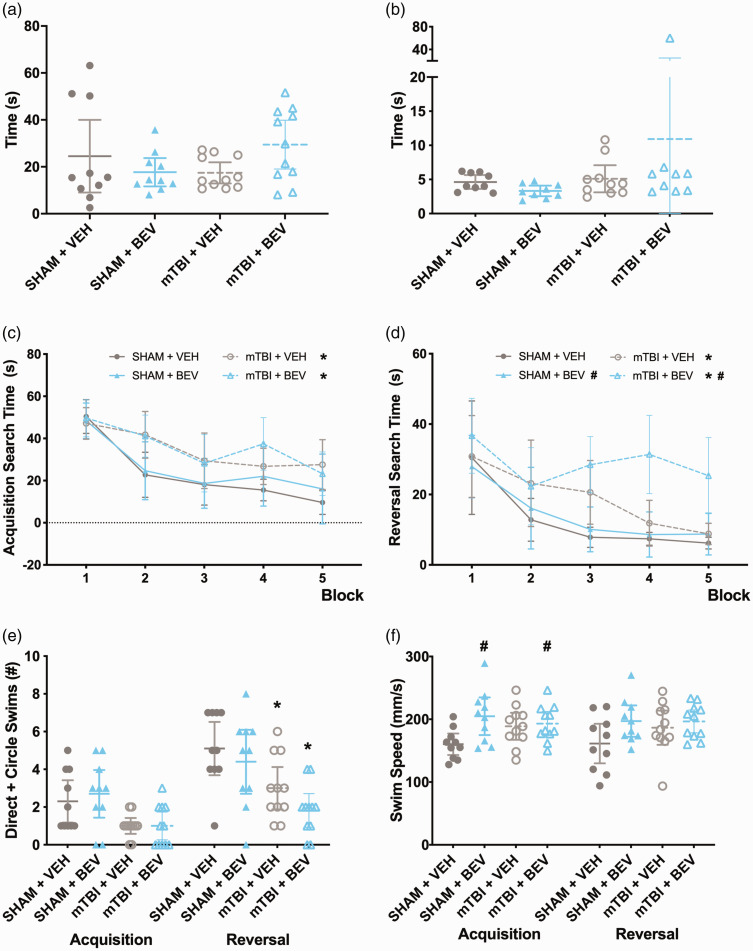
Behavior findings in male rats. No statistically significant findings were found in the open field test (a) or on the beam test (b). mTBI rats took longer to locate the platform than sham rats (c & d) during both acquisition and reversal sessions. During reversal, BEV rats took significantly longer than VEH rats to find the platform (d). mTBI rats had fewer direct and circle swims than sham rats during reversal (e). BEV rats swam faster that VEH rats regardless of mTBI/sham during acquisition, and during reversal all groups had similar swim ability (f). *mTBI significantly differed from sham, ^#^BEV significantly differed from VEH; data are presented as mean with 95% CI; block = mean of every 2 trials.

No statistically significant findings (*p* > 0.05) were found on the measure of percent time in the open arms of EPM, in the open field test (Figure 2(a)), or on the beam test (Figure 2(b)).

During water maze acquisition, there was a significant main effect for injury on the measure of search time (F_1,38_ = 15.951, *p* < 0.001; Figure 2(c)), with mTBI rats spending significantly more time to find the platform than sham rats. There was also a significant main effect for trial (F_9,342_ = 15.541, *p* < 0.001; Figure 2(c)) on the measure of search time, with rats taking less time to find the platform as testing progressed. For the measure of direct and circle swims, there was a statistically significant finding suggesting that mTBI rats had fewer direct and circle swims than shams (H (3) = 9.601, *p* < 0.05; Figure 2(e)); however post-hoc comparisons found no significant differences between the groups (*p* > 0.05). For swim speed, there was a main effect of treatment (F_1,38_ = 6.075, *p* < 0.05; Figure 2(f)), with BEV rats swimming faster that VEH rats.

During water maze reversal, there were significant main effects for injury (F_1,38_ = 15.234, *p* < 0.001; Figure 2(d)), treatment (F_1,38_ = 4.460, *p* < 0.05; Figure 2(d)), and trial (F_9,342_ = 11.996, *p* < 0.001; Figure 2(d)) on the measure of search time. Specifically, mTBI rats took significantly more time than sham rats to find the platform, BEV rats took more time than VEH rats to find the platform, and all rats improved as testing progressed. For the measure of direct and circle swims, there was a main effect of injury (F_1,38_ = 16.661, *p* < 0.001; Figure 2(e)) with mTBI rats having fewer direct and circle swims than sham rats. No statistically significant findings were found on swim speed (*p* > 0.05; Figure 2(f)).

## Gene expression findings in female rats

### Ipsilateral hippocampus

**Figure 3.**
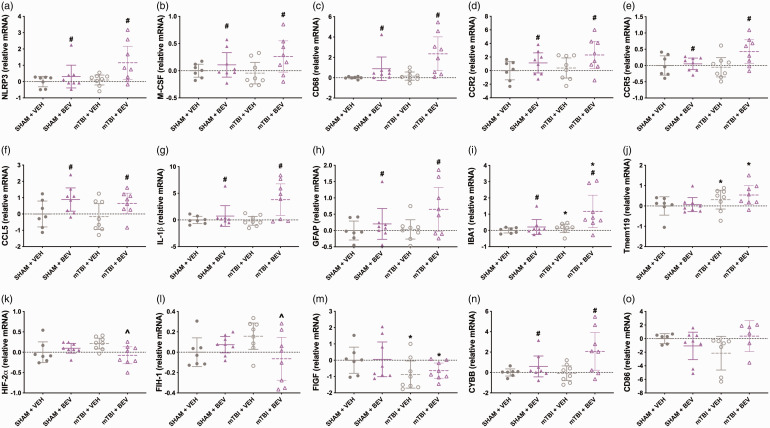
Hippocampal gene expression findings in female rats. Compared to VEH rats, BEV rats had increased expression levels of *NLRP3* (a), *M-CSF* (b), *CD68* (c), *CCR2* (d), *CCR5* (e), *CCL5* (f), *IL-1β* (g), *GFAP* (h), *IBA1* (i). mTBI rats had increased *IBA1 (i)* and *Tmem119* (j) compared to sham rats. mTBI + BEV rats had deceased expression of *HIF-2α* (k) and *FIH-1* (l) than mTBI+VEH rats. mTBI rats had declined expression of *FIGF* than sham rats (m). BEV rats had elevated expression of *CYBB* compared to VEH rats (n). No significant findings were found for *CD86* (o). *mTBI significantly differed from sham, **^#^** BEV significantly differed from VEH, **^**mTBI + BEV significantly differed from mTBI + VEH; data are presented as mean with 95% CI.

There were significant main effects for treatment on the expression of *NLRP3* (F_1,27_ = 5.787, *p* < 0.05; Figure 3(a)), *M-CSF* (F_1,27_ = 4.897, *p* < 0.05; Figure 3(b)), *CD68* (F_1,27_ = 11.485, *p* < 0.01; Figure 3(c)), *CCR2* (F_1,27_ =4.996, *p* < 0.05; Figure 3(d)), *CCR5* (F_1,27_ = 4.713, *p* < 0.05; Figure 3(e)), *CCL5* (F_1,27_ = 7.542, *p* < 0.05; Figure 3(f)), *IL-1β* (F_1,27_ = 8.497, *p* < 0.01; Figure 3(g)), *GFAP* (F_1,27_ = 4.241, *p* < 0.05; Figure 3(h)), *IBA1* (F_1,27_ = 6.277, *p* < 0.05; Figure 3(i)), with the BEV rats having elevated expression compared to VEH rats. There were significant main effects for injury on the expression of *IBA1* (F_1,27_ = 4.844, *p* < 0.05; Figure 3(i)) and *Tmem119* (F_1,27_ = 4.659, *p* < 0.05; Figure 3(j)), with mTBI rats having increased expression compared to sham rats.

There were significant injury*treatment interactions on the expression of *HIF-2α* (F_1,27_ = 6.527, *p* < 0.05; Figure 3(k)) and *FIH-1* (F_1,27_ = 5.604, *p* < 0.05; Figure 3(l)), with post-hoc analysis finding that mTBI + BEV rats had deceased *HIF-2α* and *FIH-1* when compared to mTBI+VEH rats. There was a significant main effect for injury on the expression of *FIGF* (F_1,27_ = 5.120, *p* < 0.05; Figure 3(m)), with mTBI rats having less expression compared to sham rats. There was a significant main effect for treatment on the expression of *CYBB* (F_1,27_ = 7.725, *p* < 0.05; Figure 3(n)), with the BEV rats having elevated expression compared to VEH rats. There was a significant injury*treatment interaction on the expression of *CD86* (F_1,23_ = 4.505, *p* < 0.05; Figure 3(o)), however post-hoc analysis found no significant differences between the groups. No statistically significant findings were found for the other genes tested.

### Ipsilateral temporal cortex

**Figure 4.**
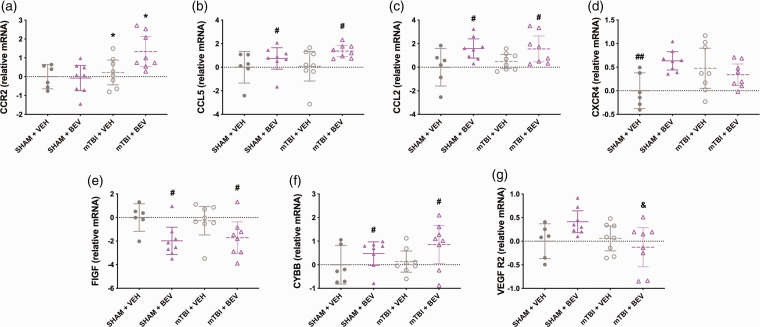
Temporal cortex gene expression findings in female rats. mTBI rats had increased *CCR2* expression compared to sham rats (a). Compared to VEH rats, BEV rats had elevated expression of *CCL5* (b) and *CCL2* (c). Sham+VEH rats had decreased expression of *CXCR4* than Sham + BEV and mTBI + VEH rats (d). BEV rats had decreased *FIGF* (e) and increased *CYBB (f)* expression compared to VEH rats. mTBI + BEV rats had decreased expression of *VEGF R2* compared to Sham + BEV rats (g). *mTBI significantly differed from sham, ^#^BEV significantly differed from VEH, ^##^significantly differed from Sham + BEV and mTBI + VEH, ^&^significantly differed from Sham + BEV; Data are presented as mean with 95% CI.

There was a significant main effect for injury on the expression of *CCR2* (F_1,26_ = 7.416, *p* < 0.05; Figure 4(a)), with mTBI rats having increased expression compared to sham rats. There was a significant main effect for treatment on the expression of *CCL5* (F_1,26_ = 5.754, *p* < 0.05; Figure 4(b)) and *CCL2* (F_1,26_ = 9.973, *p* < 0.01; Figure 4(c)), with the BEV rats having elevated expression compared to VEH rats. For *CXCR4*, there was a significant injury*treatment interaction (F_1,26_ = 8.494, *p* < 0.01; Figure 4(d)), with Sham +VEH rats having decreased levels than Sham + BEV and mTBI+VEH rats. There were significant main effects for treatment on the expression of *FIGF* (F_1,26_ = 10.761, *p* < 0.01; Figure 4(e)) and *CYBB* (F_1,26_ = 4.865, *p* < 0.05; Figure 4(f)), with the BEV rats having decreased *FIGF* and increased *CYBB* compared to VEH rats. There was a significant injury*treatment interaction on expression of *VEGF R2* (F_1,26_ = 4.810, *p* < 0.05; Figure 4(g)), with mTBI + BEV rats having decreased levels than Sham + BEV rats.

## Gene expression findings in male rats

### Ipsilateral hippocampus

**Figure 5.**
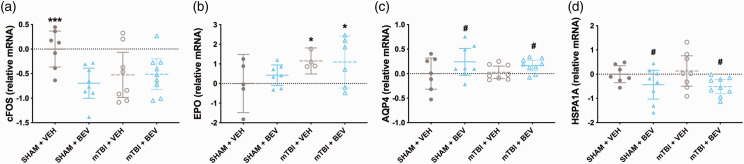
Hippocampal gene expression findings in male rats. Compared to the Sham+VEH, the Sham + BEV, mTBI + VEH, and mTBI+BEV rats had lower levels of *cFOS* expression (a). Compared to sham rats, mTBI rats had increased expression of *EPO* (b). Compared to VEH rats, BEV rats had increased *AQP4* (c) and decreased *HSPA1A* (d) expression. ***significantly differed from all other groups, *mTBI significantly differed from sham, **^#^** BEV significantly differed from VEH; data are presented as mean with 95% CI.

There was a significant injury*treatment interaction for *cFOS* (F_1,28_ = 5.251, *p* < 0.05; Figure 5(a)), with post-hoc analysis revealing that Sham + VEH rats had increased *cFOS* expression levels than all other groups. There was also a significant main effect for treatment on the expression of *cFOS* (F_1,28_ = 4.867, *p* < 0.05; Figure 5(a)). There was a significant main effect for injury on the expression of *EPO* (F_1,18_ = 4.886, *p* < 0.05; Figure 5(b)), which was increased in mTBI rats compared to sham rats. There were significant main effects for treatment on the expression of *AQP4* (F_1,28_ = 4.472, *p* < 0.05; Figure 5(c)) and *HSPA1A* (F_1,28_ = 6.822, *p* < 0.05; Figure 5(d)), with *AQP4* expression increased and *HSPA1A* decreased in BEV rats compared to VEH rats. No statistically significant findings were found for the other genes tested.

### Ipsilateral temporal cortex

**Figure 6.**
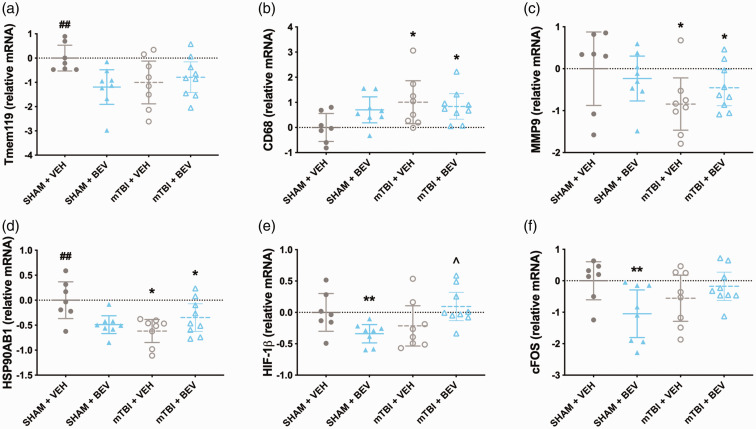
Temporal cortex gene expression findings in male rats. Sham+VEH rats had increased expression of *TMEM119* than Sham + BEV and mTBI + VEH rats (a). Compared to sham rats, mTBI rats had elevated expression of *CD68* (b), and decreased expression of *MMP9* (c) and *HSP90AB1* (d). Sham+VEH rats had increased expression levels of *HSP90AB1* compared to Sham + BEV and mTBI + VEH rats (d). Sham + BEV rats had decreased expression of *HIF-1β* compared to Sham+VEH and mTBI+BEV rats; and mTBI+BEV had increased expression of *HIF-1β* compared to mTBI + VEH (e). Sham + BEV rats had decreased expression of *cFOS* compared to Sham+VEH and mTBI+BEV rats (f). *mTBI significantly differed from sham, ** significantly differed from Sham+VEH and mTBI+BEV, ^#^BEV significantly differed from VEH, ^##^significantly differed from Sham + BEV and mTBI + VEH, ^mTBI+BEV significantly differed from mTBI + VEH; data are presented as mean with 95% CI.

There was a significant injury*treatment interaction for *TMEM119* (F_1,28_ = 5.440, *p* < 0.05; Figure 6(a)), with Sham + VEH rats having increased levels than Sham + BEV and mTBI+VEH rats. There was a significant main effect for injury on the expression of *CD68* (F_1,28_ = 4.628, *p* < 0.05; Figure 6(b)), *MMP9* (F_1,28_ = 4.284, *p* < 0.05; Figure 6(c)) and *HSP90AB1* (F_1,28_ = 4.430, *p* < 0.05; Figure 6(d)), with the mTBI rats having elevated *CD68,* and decreased *MMP9* and *HSP90AB1* compared to sham rats. There was a significant injury*treatment interaction for *HSP90AB1* (F_1,28_ = 11.341, *p* < 0.01; Figure 6(d)), *HIF-1β* (F_1,28_ =9.100, *p* < 0.01; Figure 6(e)), and *cFOS* (F_1,28_ = 6.890, *p* < 0.05; Figure 6(f)). Post-hoc analysis found that Sham + VEH rats had increased *HSP90AB1* than Sham + BEV and mTBI+VEH rats; that Sham + BEV rats had decreased *HIF-1β* and *cFOS* than Sham + VEH and mTBI + BEV rats; and that mTBI + BEV had increased levels of *HIF-1β* than mTBI+VEH.

## Discussion

This study examined the effects of continuous ICV administration of BEV for 11 days after mTBI in female and male rats. We found that BEV treatment and mTBI had adverse effects on cognitive function in the water maze in both female and male rats. Furthermore, BEV treatment and mTBI affected the expression of genes related to neuroinflammation, hypoxia, and vascular health in a sex-dependent manner. In females, BEV treatment significantly decreased the expression of hippocampal *HIF-2α* and *FIH-1* in mTBI rats; BEV treatment increased the expression of the inflammatory markers *NLRP3*, *M-CSF*, *CD68*, *CCR2*, *CCR5*, *CCL5, IL-1β, GFAP*, *and IBA1* in the hippocampus, as well as *CCL2* and *CCL5* in the temporal cortex;^50,51^ BEV treatment significantly increased the expression of *CYBB* (also known as *NOX2*) and decreased the expression of *FIGF* in the temporal cortex; and mTBI increased the levels of the inflammatory markers *IBA1* and *Tmem119* in the hippocampus and *CCR2* in the temporal cortex. In males, BEV treatment increased *HIF-1β* levels in the temporal cortex of mTBI rats, both BEV treatment and mTBI decreased hippocampal *cFOS* gene expression (i.e., an immediate early gene associated with hypoxia, angiogenesis, and cellular proliferation, differentiation, and survival);^52^ BEV treatment increased expression of *AQP4* and decreased expression of *HSPA1A* in the hippocampus; and mTBI increased expression of hippocampal *EPO* (i.e., a cytokine that stimulates erythropoiesis and angiogenesis),^53^ increased *CD68* in the temporal cortex, and decreased expression of *MMP9 and HSP90AB1* in the temporal cortex.

## Insights into the role of VEGF-A in mTBI recovery

VEGF-A is a potent promoter of angiogenesis and an important modulator of vascular health, and previous studies suggest an active role of VEGF-A in the response to mTBI. Elevated VEGF has been reported in the blood of mTBI patients and athletes exposed to concussive and sub-concussive brain injuries,^50,51^ as well as in the CSF and brain tissue from rodents given mTBI.^54,55^ Evidence in more severe TBI suggests that increased bioavailability of VEGF-A is neuroprotective and promotes cerebral vascular repair,^26^ neuronal survival, and neurogenesis,^25^ whilst reducing contusion necrosis and neurological deficits.^28^ However, few studies have investigated how VEGF-A influences mTBI recovery. As such, we recently examined the effects of recombinant VEGF-A treatment in rats given a mTBI using the same intervention regimen as the present study (i.e., continuous ICV administration from the time of injury to the end of the study).^32^ VEGF-A treatment resulted in worse cognitive recovery in male mTBI rats and in both mTBI and sham female rats. VEGF-A treatment also affected gene expression on neuroinflammation and hypoxia related markers in a sex-specific manner.^32^ Combined with the results from the present study, our collective findings indicate that ICV administration of either pro- or anti-VEGF-A agents during acute and sub-acute mTBI recovery has minimal or detrimental effects depending on sex and suggests that an endogenous VEGF-A response is favorable over exogenous modification.

## The influence of BEV treatment on gene expression

*cFOS* is an immediate early gene in response to hypoxia-ischemia injury; however whether it promotes neuronal death or survival during brain injury is unclear and previous studies have reported conflicting results.^52^ In males, we observed a decrease of hippocampal *cFOS* 11 days after mTBI and continuous BEV treatment. BEV treatment also upregulated gene expression levels of *AQP4* in the hippocampus, an important mediator of edema, in male rats. Previous studies have found that decreasing *AQP4* levels after TBI mitigates brain edema and hippocampal damage.^56^ Considering that BEV treatment also negatively affected cognition in male rats, and the central role of the hippocampus in this task, it is possible that the influence of BEV on hippocampal *cFOS* and *AQP4* contributed to these findings. Furthermore, the lack of BEV-induced changes on hippocampal *cFOS* and *AQP4* in females may explain the observation that BEV did not negatively affect cognition in female rats.

BEV treatment did however influence the expression of other genes in female rats, including the upregulation of inflammatory markers *NLRP3*, *M-CSF*, *CD68*, *CCR2*, *CCR5*, *CCL5, IL-1β, GFAP*, and *IBA1* in the hippocampus, as well as *CCL2, CCL5,* and *CYBB* (also known as *NOX2* and involved in the generation of oxygen reactive species and promoting neuroinflammation)^57–60^ in the temporal cortex. This evident increase in neuroinflammation in both brain regions examined was associated with corresponding behavioural impairments in the BEV-treated female rats (e.g., mTBI + BEV had fewer direct and circle swims in the water maze than the Sham + VEH). BEV treatment also decreased the gene expression levels of hippocampal *HIF-2α* and *FIH-1* in females given a mTBI. The HIFs are a family of proteins that modulate cellular adaptation in response to reduced cerebral oxygen and maintain cell homeostasis.^61,62^ FIH-1 inhibits HIF-1, blocking the transcriptional activity of *HIF-1* under normoxic conditions.^63,64^ Taken together, these findings suggest different effects of BEV treatment on gene regulation in female and male rats.

## Limitations & future directions

Interpretations and conclusions should be limited to the specific treatment paradigm, including dose, administration methods, and time window, that was tested. For example, previous studies that report beneficial effects of exercise, which is known to upregulate VEGF-A,^30,31^ after mTBI typically do not initiate the exercise intervention until days or weeks after the injury.^29,48,65,66^ Additionally, the gene expression analysis conducted at day 11 post-injury may have missed other peak physiological responses to both injury and treatment. Therefore, including additional post-mortem timepoints and analyzing other brain regions could provide a better understanding of how BEV treatment influences mTBI pathophysiology. A further limitation is that our pathophysiological studies were limited to gene expression analysis, and it would have been informative to conduct an analysis of protein expression as well as immunohistological investigations. In humans, BEV treatment has potential side effects such as increased blood pressure, faintness, headache, confusion, and seizures, which may have influenced our findings.^67–69^ Although ICV administration of drugs are appropriate for this proof-of-rational study, more clinically relevant drug delivery methods should be considered for future studies. A strength of our study was the inclusion of both male and female rats so that we could examine the impact of BEV and mTBI within each of the sexes. Although our findings provide initial indirect evidence that some of the effects of BEV and mTBI may be sex-dependent, future studies that are appropriately designed and statically powered to directly compare males and females are required. Finally, this study was largely associative in nature and further investigations into cause-and-effect relationships between patterns of gene expression, vascular and immune related events, and functional outcomes would be informative.

## Conclusions

ICV treatment with BEV, an anti-VEGF-A monoclonal antibody, had negative effects on female and male rats, resulting in worse cognitive function that was accompanied by sex-specific gene expression changes related to hypoxia, edema, and vascular health. When combined with our previous findings that continuous ICV treatment with recombinant VEGF-A negatively affected mTBI recovery, our results suggest that exogenously modifying VEGF-A availability and activity does not improve mTBI recovery, irrespective of sex. Taken together, our findings offered important insights related to VEGF-A and mTBI in both males and females.

## Supplemental Material

sj-pdf-1-jcb-10.1177_0271678X231212377 - Supplemental material for Treatment with the vascular endothelial growth factor-A antibody, bevacizumab, has sex-specific effects in a rat model of mild traumatic brain injury Supplemental material, sj-pdf-1-jcb-10.1177_0271678X231212377 for Treatment with the vascular endothelial growth factor-A antibody, bevacizumab, has sex-specific effects in a rat model of mild traumatic brain injury by Mujun Sun, Tamara L Baker, Campbell T Wilson, Rhys D Brady, Glenn R Yamakawa, David K Wright, Richelle Mychasiuk, Anh Vo, Trevor Wilson, Josh Allen, Stuart J McDonald and Sandy R Shultz in Journal of Cerebral Blood Flow & Metabolism
