# Supplementary material for: Treatment with the vascular endothelial growth factor-A antibody, bevacizumab, has sex-specific effects in a rat model of mild traumatic brain injury
Source: J Cereb Blood Flow Metab. 2023 Nov 7;44(4):542–55. doi: 10.1177/0271678X231212377 (PMC10981407; doi:10.1177/0271678X231212377)
Supplement: sj-pdf-1-jcb-10.1177_0271678X231212377 - Supplemental material for Treatment with the vascular endothelial growth factor-A antibody, bevacizumab, has sex-specific effects in a rat model of mild traumatic brain injury [file sj-pdf-1-jcb-10.1177_0271678X231212377.pdf]

Supplementary Table 1. The TaqMan® gene expression assays. Gene expression assays related to neuroinflammation, hypoxia injury and vascular health, and 4 housekeeping gene assays were used.

Gene	Thermo Fisher Assay
	Vascular health
VEGF-A	Rn01511602_m1
MMP9	Rn00579162_m1
MMP2	Rn01538169_m1
S1P1	Rn02758712_s1
VEGF R2	Rn00564986_m1
AQP4	Rn01401322_m1
TGFB1	Rn00572010_m1
	Neuroinflammation
TMEM119	Rn01480631_m1
M-CSF	Rn01522726_m1
IL-6	Rn01410330_m1
TNF	Rn99999017_m1
IL-1α	Rn00566700_m1
IL-1β	Rn00580432_m1
IFNG	Rn99999014_m1
CCL2	Rn00580555_m1
CCL5	Rn00579590_m1
CXCR4	Rn01483207_m1
IBA1	Rn00567906_g1
GFAP	Rn00566603_m1
CD68	Rn01495634_g1
CD86	Rn00571654_m1
CCR5	Rn00588629_m1
CCR2	Rn01637698_s1
NLRP3	Rn04244622_m1
iNOS	Rn00561646_m1
	Hypoxia
HIF-1α	Rn01642006_m1
HIF-2α	Rn00576515_m1
HIF-1β	Rn00688999_m1
FIH-1	Rn01766292_m1
EPO	Rn01481376_m1
nNOS	Rn00583793_m1
eNOS	Rn07312037_g1
cFOS	Rn02396759_m1
FIGF	Rn00582193_m1
GLUT1	Rn01417099_m1
	Cell stress
HSP90AB1	Rn01511686_g1

HSP27	Rn00583001_g1
HSPA1A	Rn04224718_u1
HSF1	Rn00801772_m1
Others	
SDHA	Rn00590475_m1
CASP9	Rn00581212_m1
CYBB	Rn00576710_m1
Housekeeping genes	
HPRT	Rn01527840_m1
B2M	Rn00560865_m1
YWHAZ	Rn00755072_m1
PPIA	Rn00690933_m1
